# Myelin-specific multiple sclerosis antibodies cause complement-dependent oligodendrocyte loss and demyelination

**DOI:** 10.1186/s40478-017-0428-6

**Published:** 2017-03-24

**Authors:** Yiting Liu, Katherine S. Given, Danielle E. Harlow, Adeline M. Matschulat, Wendy B. Macklin, Jeffrey L. Bennett, Gregory P. Owens

**Affiliations:** 1 Department of Neurology, University of Colorado, School of Medicine, 12700 E. 19th Ave, Aurora, CO USA; 2 Department of Cell and Developmental Biology, University of Colorado, School of Medicine, 12700 E. 19th Ave, Aurora, CO USA; 3 Department of Ophthalmology, University of Colorado, School of Medicine, 12700 E. 19th Ave, Aurora, CO USA; 4 Program in Neuroscience, University of Colorado, School of Medicine, 12700 E. 19th Ave, Aurora, CO USA

**Keywords:** Antibodies, Complement, Cytotoxicity, Myelin, Oligodendrocytes, Demyelination

## Abstract

Intrathecal immunoglobulin G (IgG) synthesis, cerebrospinal fluid (CSF) oligoclonal IgG bands and lesional IgG deposition are seminal features of multiple sclerosis (MS) disease pathology. Both the specific targets and pathogenic effects of MS antibodies remain poorly characterized. We produced IgG1 monoclonal recombinant antibodies (rAbs) from clonally-expanded plasmablasts recovered from MS patient CSF. Among these were a subset of myelin-specific MS rAbs. We examined their immunoreactivity to mouse organotypic cerebellar slices by live binding and evaluated tissue injury in the presence and absence of human complement. Demyelination, glial and neuronal viability, and complement pathway activation were assayed by immunofluorescence microscopy and compared to the effects of an aquaporin-4 water channel (AQP4)-specific rAb derived from a neuromyelitis optica (NMO) patient. MS myelin-specific rAbs bound to discrete surface domains on oligodendrocyte processes and myelinating axons. Myelin-specific MS rAbs initiated complement-dependent cytotoxicity to oligodendrocytes and induced rapid demyelination. Demyelination was accompanied by increased microglia activation; however, the morphology and survival of astrocytes, oligodendrocyte progenitors and neurons remained unaffected. In contrast, NMO AQP4-specific rAb initiated complement-dependent astrocyte damage, followed by sequential loss of oligodendrocytes, demyelination, microglia activation and neuronal death. Myelin-specific MS antibodies cause oligodendrocyte loss and demyelination in organotypic cerebellar slices, which are distinct from AQP4-targeted pathology, and display seminal features of active MS lesions. Myelin-specific antibodies may play an active role in MS lesion formation through complement-dependent mechanisms.

**Electronic supplementary material:**

The online version of this article (doi:10.1186/s40478-017-0428-6) contains supplementary material, which is available to authorized users.

## Introduction

Multiple sclerosis (MS) is a chronic inflammatory demyelinating disease of the central nervous system (CNS) of unknown cause. Despite extensive pathological characterization of heterogeneous MS lesions, no consensus on the cellular and molecular mechanisms driving diverse lesion pathology has emerged [15, 24, 30]. The presence of persistent cerebrospinal fluid (CSF) oligoclonal immunoglobulin G (IgG) bands produced by intrathecal IgG synthesis in MS patients is one of the most striking biochemical hallmarks of disease [21]. Deposition of IgG and activated complement products are present in the most frequently seen Type II MS lesions [15], suggesting a possible role of intrathecal IgG in CNS tissue injury.

We have constructed recombinant monoclonal IgG1 antibodies (rAbs) from expanded CSF plasmablast clones isolated from MS patients [22] and demonstrated their differential patterns of binding to antigens expressed by astrocytes and neurons or to myelin-enriched antigens [3, 13]. In cDNA-transfected HEK cells or by protein immunoblotting of human brain lysate, myelin-specific rAbs failed to recognize myelin-enriched proteins, including myelin basic protein (MBP), proteolipid protein (PLP) and myelin oligodendrocyte glycoprotein (MOG) [22], and their specific targets remain elusive. Nevertheless, both myelin and neuron/astrocyte-targeted MS rAbs cause myelin loss when applied to mouse spinal cord explant cultures in the presence of human complement [3], indicating that, similar to autoantibodies against aquaporin-4 (AQP4-IgG) in neuromyelitis optica (NMO) [2, 4, 28], intrathecal IgGs in MS may contribute to lesion pathogenesis.

In this study, we further investigated the primary effect of myelin-specific MS rAbs on intact CNS tissue using organotypic mouse cerebellar slice cultures. Our results reveal that MS myelin-specific rAbs recognized surface antigens on oligodendrocyte processes and the outer layer of myelin ensheathing axons. In the presence of human complement, these rAbs initiated classical complement pathway activation leading to oligodendrocyte death and rapid demyelination. The extent and timing of glial and neuronal injury was distinct from damage driven by AQP4-IgG and reproduced some hallmark features of MS lesions, further distinguishing MS from NMO and supporting an active role for intrathecal MS IgG in CNS lesion formation.

## Materials and methods

### Animals

The care and euthanasia of animals were in accordance with University of Colorado IUCAC policy for animal use, which is in agreement with the NIH Guide for the Care and Use of Laboratory Animals.

### Recombinant antibodies

Myelin-specific MS monoclonal recombinant antibodies (rAbs) used in this study were constructed from expanded CSF plasma blast clones derived from a relapsing-progressive MS patient 6 months after disease onset (MS04-2, for rAb MS04-2#30 [22] or from an isolated optic neuritis patient following their first clinical event who subsequently progressed to clinically-definite MS (ON07-7, for rAb MS07-7#49). NMO monoclonal recombinant anti-AQP4 antibody #53 (NMO#53) was cloned from seropositive NMO patient CSF plasma blast [2], and the isotype-control human antibody (Iso) was generated from a chronic meningitis patient CSF plasma blast clone [3]. All rAbs were expressed as full-length bivalent human IgG1 antibodies containing a C-terminal Flag epitope using a dual vector transient transfection system and purified with protein A-sepharose (Sigma-Aldrich, St. Louis, MO) as previously described [2, 22]. All rAbs were used at 20 μg/ml in the slice cultures.

### Cerebellar slice culture

Sagittal cerebellar slices (300 μm) were prepared from PLP-eGFP mice [18] at P10 and cultured on MilliCell 0.4 μm membrane inserts (Millipore, Billerica, MA) for 10–14 days in slice media (25% Hank’s balanced salt solution (HBSS), 25% heat-inactivated horse serum, 50% minimum essential media (MEM), 125 mM HEPES, 28 mM D-Glucose, 2 mM L-Glutamine, 10U/ml penicillin/streptomycin, all from Life Technologies, Carlsbad, CA) at 37 °C [29]. Prior to treatment, slices were switched to a serum-free media (Neurobasal medium supplemented with B27, 2 mM L-glutamine, 10U/ml penicillin/streptomycin and 28 mM D-glucose).

### Treatment of cerebellar slices

rAbs were applied at 20 μg/ml with or without 10% normal or C5-depleted human serum (Complement Technology, Tyler, TX). Media containing treatment reagents were applied both on top (50 μl) and below (250 μl) the membrane insert. For live binding assays, unfixed slices were incubated with 20 μg/ml rAbs for 4 h at 37 °C. Propidium iodide (PI) (Sigma) was used at 5 μg/ml in the culture medium to label dead cells in organotypic slice cultures. Normal and C5-depleted human serum (Complement Technology, Tyler, TX) were used at 10% (vol/vol) as the source of human complement (HC).

### Tissue preparation

For immunostaining, adult C57bl/6 mice were perfused with 4% paraformaldehyde in phosphate-buffered saline (PBS), and the brain was subsequently removed, post-fixed overnight in 4% paraformaldehyde, and cryoprotected overnight in 20% sucrose at 4 °C. Mouse brain was embedded in optimal cutting temperature (OCT) freezing media, and 6–10 μm cryostat sections collected on Superfrost Plus microscope slides (Fisher Scientific, Pittsburgh, PA). Tissue sections were stored at −80 °C until immunostaining.

### Immunostaining

After treatment, cerebellar slices were rinsed twice with PBS and fixed in 4% paraformaldehyde in PBS for 20 min at 4 °C. For immunohistochemistry, slices were rinsed in PBS and permeabilized in 1.5 or 10% (myelin proteins) Triton X-100 in PBS for 20 min. Slices were rinsed, blocked with 5% normal donkey serum (NDS) in PBS with 0.3% Triton X-100 for 1 h, and incubated with primary antibodies overnight at room temperature. Following 3 washes in PBS, Alexa Fluor-labeled secondary antibodies (Jackson ImmunoResearch, West Grove, PA) were applied (1:800) overnight at room temperature, washed 3 times in PBS and mounted in Fluoromount G (Southern Biotech, Birmingham, AL). The following primary antibodies were used: rabbit anti-GFAP (Sigma), rabbit anti-Calbindin (Millipore), mouse anti-MAG (Millipore), rabbit anti-MOG (Abcam, Cambridge, United Kingdom), mouse anti-MBP (Covance, Princeton, NJ), chicken anti- Neurofilament-H (Neuromics, Minneapolis, MN), goat anti-Iba1 (Abcam), mouse anti-C3d (a gift from Dr. Joshua Thurman, University of Colorado), rabbit anti-MAC complex (anti C5b-9, Abcam), guinea pig anti- NG2 and rabbit anti-Olig2 (are gifts from Dr. Charles Stiles, Harvard University).

Prior to immunostaining, PFA-fixed mouse cerebellum tissue sections were thawed for 10 min, re-hydrated in PBS for 10 min, and blocked in PBS containing 3% bovine serum albumin (BSA), 2% normal goat serum (NGS), and 0.3% Triton-X100 for 1 h. MS rAbs were applied at a final concentration of 20 μg/mL to mouse tissues for 16 h at 4 °C in PBS containing 3% BSA and 2% NGS. Tissue sections were then washed 3 times for 3 min with PBS. Alexa fluorescent secondary antibody (1:1000) against human IgG (Molecular probes, Life Science Technologies) was applied for 1 h at room temperature in PBS containing 3% BSA and 2% NGS. Tissue was then washed 5 times for 3 min in PBS and mounted using Vectashield (Vector Laboratories, Burlingame, CA, USA) containing DAPI.

### Microscopy

Fluorescence images were acquired by Zeiss fluorescence microscope with Axiovision software (Zeiss, Jena, Germany). Confocal images were acquired by Leica SP5 laser scanning microscope (Leica Microsystems GmbH, Wetzlar, Germany). Super-resolution structured illumination microscopy was performed using Nikon’s N-SIM (Nikon, Tokyo, Japan).

### Quantification and statistical analysis

PLP-eGFP positive cell bodies in the cerebellar slices were imaged using a Zeiss fluorescence microscope with 20X objective. Images were quantified with ImageJ (National Institutes of Health open source). To quantify the extent of myelination, we calculated the percentage of total neurofilament-H staining that was co-labeled with MBP using a Matlab algorithm (MathWorks, Natick, MA). For each slice, 2–3 images were taken, quantified, and averaged. Slices from 3–4 independent experiments were analyzed. Statistical analyses were performed by unpaired Student’s *t* test for single comparisons or by two-way ANOVA for grouped comparisons using GraphPad Prism software. Data are expressed as means ± SD of independent experiments (*n* ≥ 3). Significance is reported for *p* < 0.05.

## Results

### Myelin-specific MS rAb binds to oligodendrocyte processes and myelinated axons

**Fig. 1:**
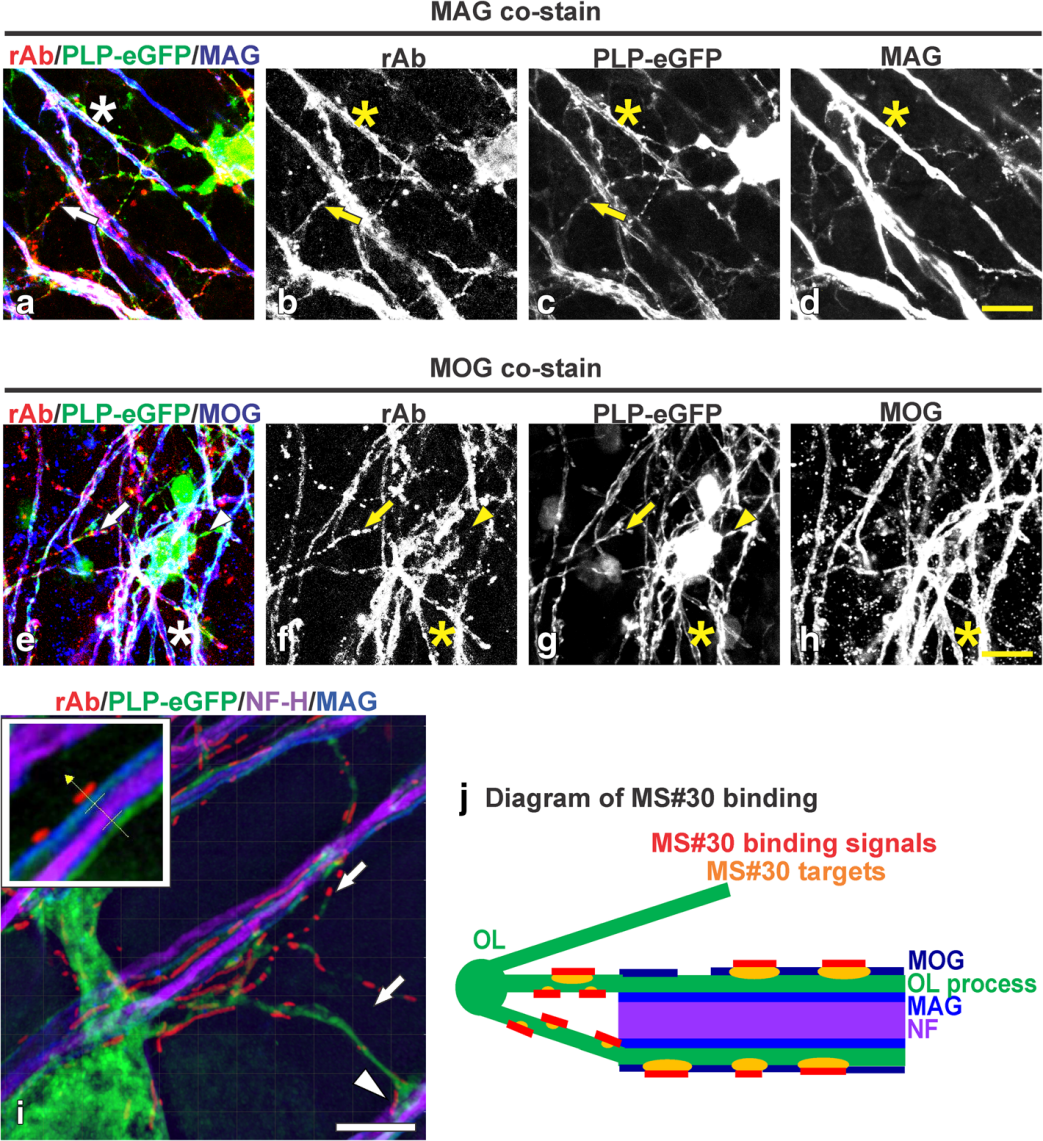
MS rAb #30 (MS#30) binds to oligodendrocyte processes and myelinated axons. Live cerebellar slices prepared from PLP-eGFP mouse pups were incubated with 20 μg/ml MS#30 rAb (red), then fixed and immunostained with anti-human IgG secondary and the indicated myelin markers. Confocal images of slices co-stained for the myelin proteins MAG (**a**–**d**; *blue*) and MOG (**e**–**h**; *blue*). MS#30 staining was co-localized with PLP-eGFP+ oligodendrocyte processes (*arrows*) and MAG+ or MOG+ myelinated axons (*stars*). Not all oligodendrocyte processes were stained with MS#30 (*arrow heads*). *Scale bars*: 10 μm. Super-resolution structured illumination microscopy image (**i**) of live slices stained with MS#30 (*red*), then fixed and stained for MAG (*blue*) and NF-H (*purple*). MS#30 reactivity was on oligodendrocyte processes (*arrows*), including those contacting adjacent axons (*arrow head*), and on myelinated MAG+ axons, outside of MAG layer (*insert*). *Scale bar*: 2 μm. Diagram of MS#30 rAb binding pattern (**j**)


We examined the live binding pattern of the myelin-specific MS rAb, MS04-2 #30 (MS#30) [3] in mouse organotypic cerebellar slice cultures. MS#30 rAb co-localized with myelin-associated glycoprotein-positive (MAG+) or myelin oligodendrocyte glycoprotein-positive (MOG+) axons (co-stained with neurofilament-H [NF-H], data not shown) (Fig. 1a–h; stars), as well as enhanced green fluorescent protein-positive (eGFP+) oligodendrocyte processes (Fig. 1a–h; arrows), although some oligodendrocyte processes did not bind this antibody (Fig. 1e–g; arrow heads). MS#30 reactivity was primarily located in discrete surface domains external to MAG on myelinated axons or along oligodendroglial processes projecting to axons with little to no surface staining of oligodendrocyte cell bodies (Fig. 1i; arrows and arrow heads; Online Resource, Additional file 1: Video S1). The binding pattern suggests that MS#30 recognizes an antigen produced and transported by oligodendrocytes to the myelin sheath (Fig. 1j).

Additional file 1: Video S1. 3D movie reconstructed by super-resolution structured illumination microscopy (SIM) imaging of live organotypic mouse cerebellar slices stained with MS#30 (red), then fixed and stained for MAG (blue) and NF-H (purple). MS#30 reactivity was on oligodendrocyte processes, including those contacting adjacent axons, and on myelinated MAG+ axons, outside of MAG layer. Scale bar: 2 μm. (MPG 90340 kb)


### MS#30 causes loss of mature oligodendrocytes in the presence of human complement

**Fig. 2:**
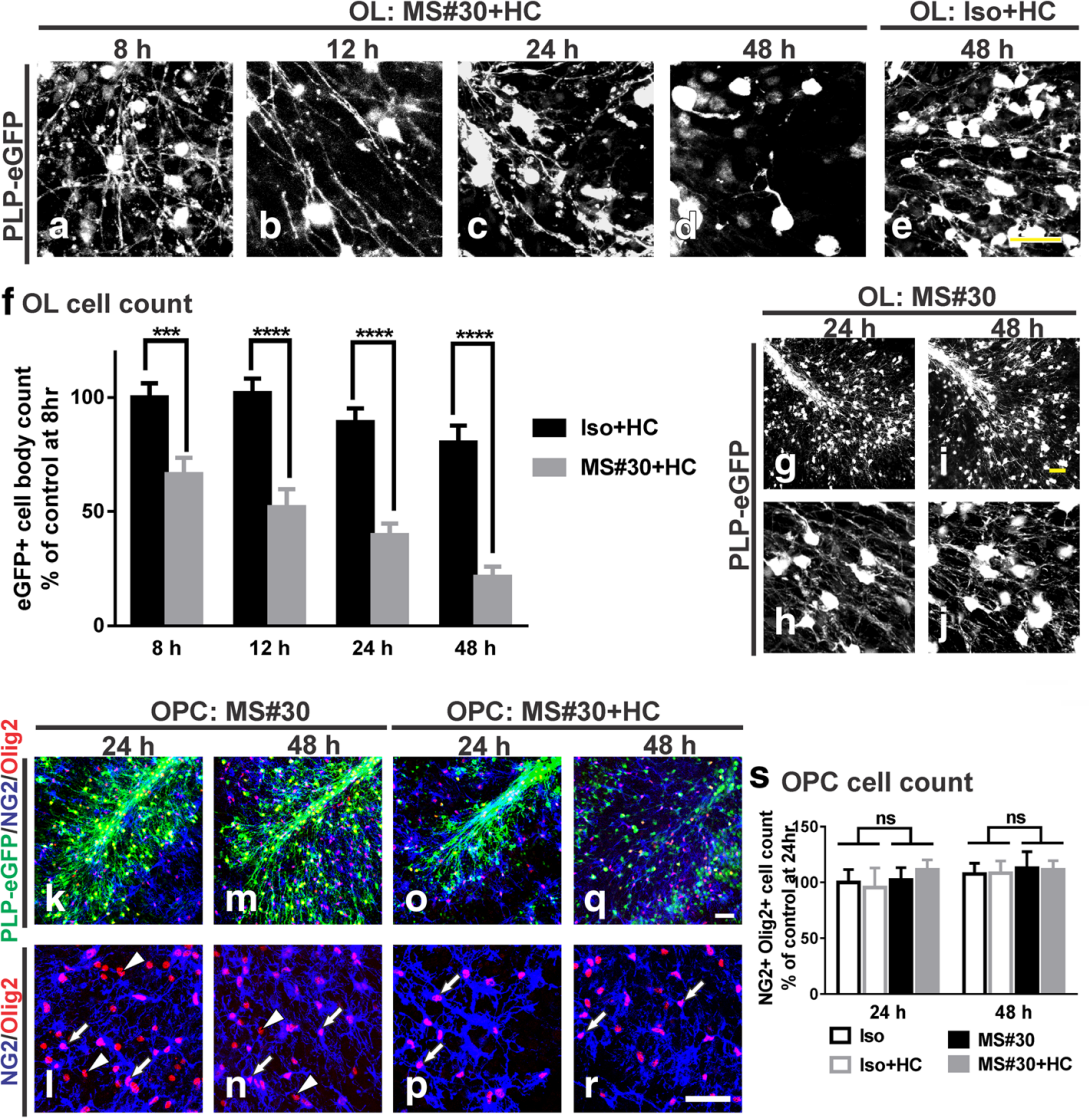
MS#30 plus human complement (HC) causes robust loss of mature oligodendrocytes but not progenitors. Confocal images of PLP-eGFP in slices treated with 20 μg/ml MS#30 plus 10% (vol/vol) HC for 8 (**a**), 12 (**b**), 24 (**c**) and 48 h (**d**) and isotype control rAb (Iso) + HC for 48 h (**e**). Quantification of eGFP+ oligodendrocyte cell bodies in slices (**f**). Cell body count was normalized to control (*Iso + HC treated*) slice at 8 h. 25X (**g**, **i**) and 40X (**h**, **j**) objective confocal images of PLP-eGFP in slices treated with MS#30 alone for 24 and 48 h. 25X (**k**, **m**, **o**, **q**) and 40X (**l**, **n**, **p**, **r**) objective confocal images of PLP-eGFP slices treated with MS#30 alone or MS#30 + HC at indicated time and co-stained with Olig2 (*red*) and NG2 (*blue*). Oligodendrocyte progenitors were identified as Olig2+ NG2+ cells (*arrows*). Olig2+ NG2- cells were mature oligodendrocytes (*arrow heads*) and overlapped with PLP-eGFP (25X *objective panels*). Quantification of NG2+ Olig2+ oligodendrocyte progenitor cell number in slices (**s**). Cell count was normalized to control (*Iso treated*) slice at 24 h. Statistical analyses were performed by multiple unpaired Student’s *t* test for single comparison (**f**) or by two-way ANOVA for grouped comparisons (**s**). ***: *p* < 0.001, ****: *p* < 0.0001, ns: not significant, *n* = 4. Scale bars: 50 μm

We next examined the effects of MS#30 on *ex vivo* cerebellar slices. Treatment with MS#30 plus human complement (HC) resulted in altered oligodendrocyte morphology and viability. As early as 8 h, oligodendrocyte processes were disorganized and fragmented (Fig. 2a). By 48 h, there was complete loss of oligodendrocyte processes, and the few remaining cell bodies were hypertrophic and devoid of extended processes (Fig. 2d). PLP-eGFP+ oligodendrocyte cell bodies diminished with continued exposure to MS#30 + HC (Fig. 2a–d). At 8 h, we observed a 33.6 ± 7.2% loss compared to isotype control rAb (Iso) plus HC (*p* < 0.001), and by 48 h, eGFP+ oligodendrocyte cell bodies were reduced by 73.1 ± 4.4% (*p* < 0.0001). By comparison, there was a minor, but statistically significant loss (about 10%) of oligodendrocyte cells following treatment with Iso + HC from 24 h (Fig. 2e, f), which is consistent with our previous finding that HC causes some modest oligodendrocyte loss in cerebellar slices after 24 h treatment [14]. No changes in oligodendrocyte morphology or viability were noted following treatment with MS#30 in the absence of HC with preservation of 95.8 ± 2.7 and 101.9 ± 16.5% of eGFP+ oligodendrocyte cell bodies respectively at 24 h and 48 h when compared to Iso treatment (Fig. 2g–j and data not shown). Although MS#30 + HC caused significant damage to the mature oligodendrocyte population as evidenced by loss of eGFP+ cell bodies and processes, the oligodendrocyte progenitor population was unaffected. Neither treatment with MS#30 or MS#30 + HC induced changes to the morphology or cell numbers of NG2+/Olig2+ (neural/glial antigen 2; oligodendrocyte transcription factor 2) progenitors at 24 h and 48 h (Fig. 2k–s).

### MS#30-mediated demyelination is distinct from AQP4-targeted demyelination

**Fig. 3:**
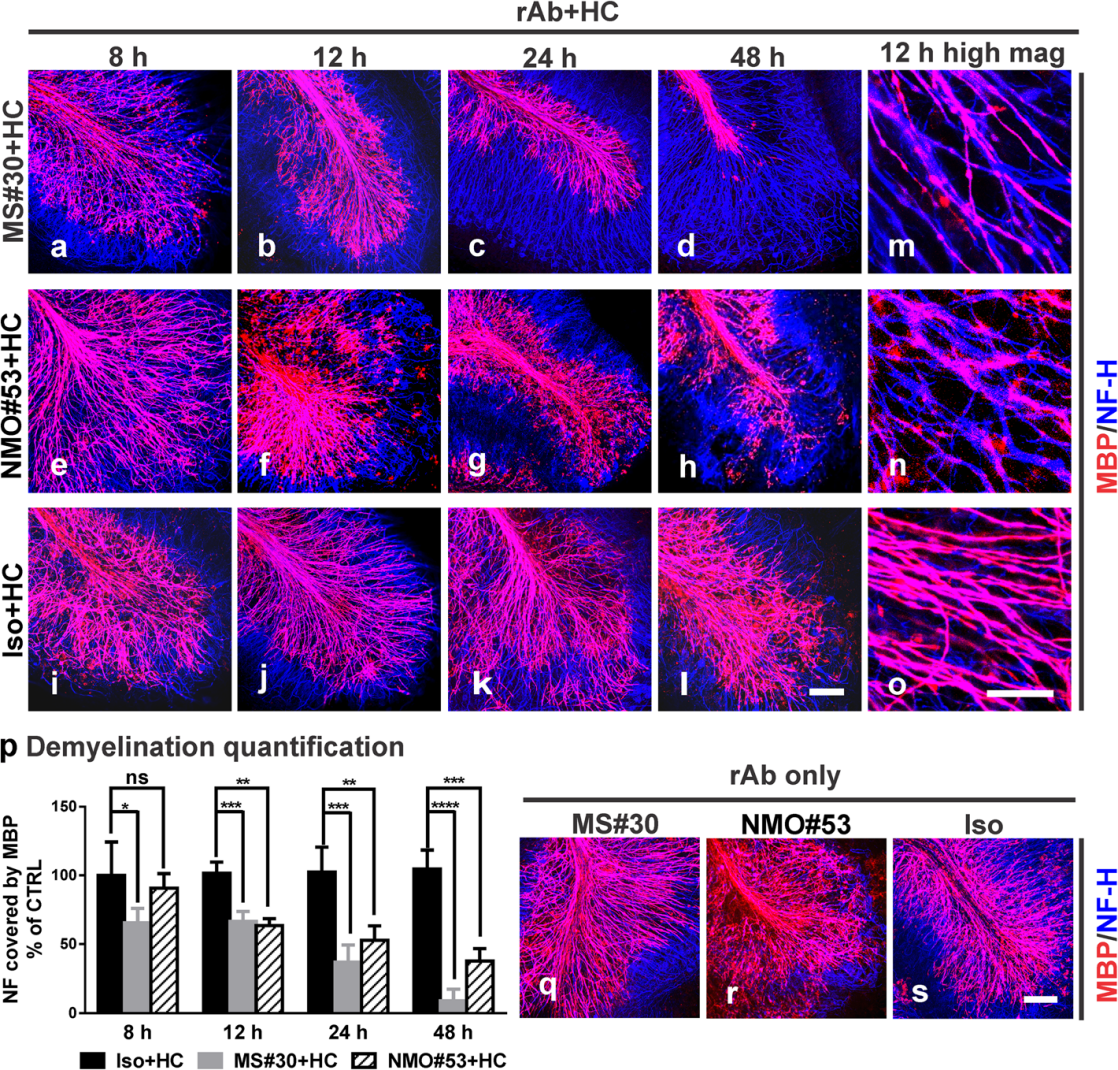
MS#30 and NMO rAb #53 (NMO#53) induce demyelination at different rates. Cerebellar slices were treated with MS#30 + HC (**a**–**d**, **m**), or NMO#53 + HC (**e**–**h**, **n**), or Iso + HC (**i**–**l**, **o**). At the indicated time points, slices were fixed and stained with MBP (*red*) and NF-H (*blue*) antibodies. Confocal images were taken with 25X (**a**–**l**) or 63X (**m**–**o**) objectives. The coverage of MBP on NF-H+ axons was quantified using a Matlab algorithm and normalized by control (*Iso + HC treated slices*) at 8 h (**p**). Statistical analyses were performed by multiple unpaired Student’s *t* test. *: *p* < 0.05, **: *p* < 0.01, ***: *p* < 0.001, ****: *p* < 0.0001, ns: not significant, *n* = 3-4. Confocal images of MBP and NF-H stained slices treated with rAbs alone at 48 h (**q**–**s**). *Scale bars*: 100 μm

MS and NMO are both CNS inflammatory diseases characterized by myelin loss. We next compared the pattern and extent of MS#30 CNS demyelination with that caused by treatment with a pathogenic AQP4-specific rAb, NMO#53, which was derived from an expanded plasma blast clone isolated from NMO IgG-positive CSF [2]. Concomitant with oligodendrocyte cell loss, MS#30 + HC treatment showed a rapid and progressive loss of myelin basic protein (MBP) along axons that was first observed at 8 h after exposure (Fig. 3a–d, m; compare 3a and i). Slices treated with NMO#53 + HC also exhibited progressive myelin loss beginning at 12 h (Fig. 3e–h, n; compare 3f and j) with MBP staining displaying a distinct patchy and ‘debris-like’ pattern. Demyelination under this scenario follows complement-dependent destruction of astrocytes and is accompanied by significant oligodendrocyte cell loss [13]. There were no measurable effects on MBP staining of slices treated with Iso + HC or HC alone (Fig. 3i–l, o and data not shown). To assess the extent of cerebellar demyelination, we quantified the percent of MBP-covered NF-H+ axons. Treatment with MS#30 + HC caused a significant 34.5 ± 10.6% loss of MBP at 8 h (*p* < 0.05) that increased to a 91.0 ± 8.6% loss at 48 h (*p* < 0.0001). Demyelination occurred later in slices treated with NMO#53 + HC, with a 36.6 ± 5.1% loss beginning at 12 h (*p* < 0.01), and reaching 62.3 ± 9.0% (*p* < 0.001) MBP loss at 48 h (Fig. 3p). In the absence of HC, neither MS#30 nor NMO#53 induced demyelination (Fig. 3q–s).

### Demyelination by MS#30 plus HC results in microglia activation, but not astrocyte or neuronal loss

**Fig. 4:**
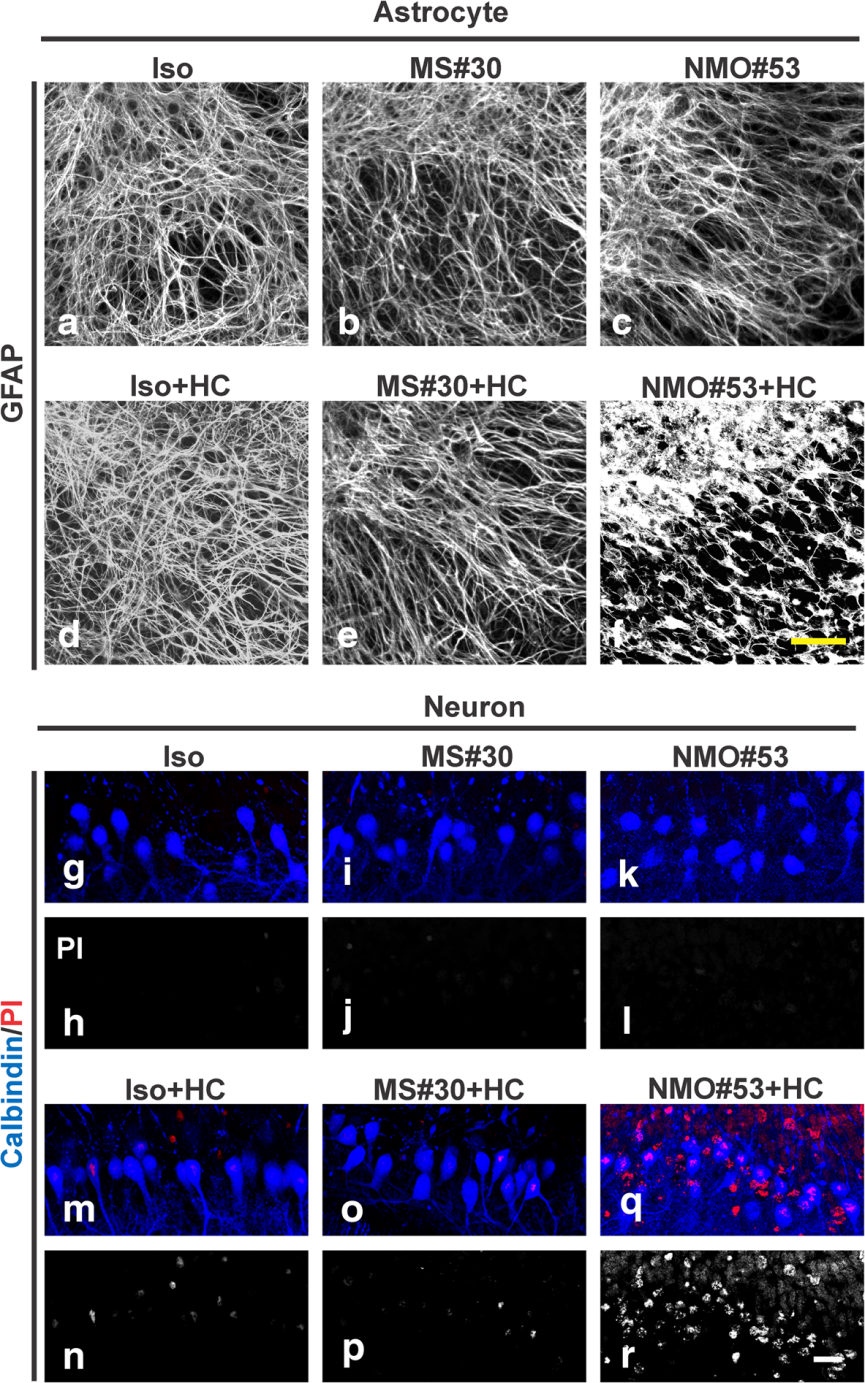
MS#30 and NMO#53 have distinct effects on astrocytes and neurons. Immunostaining of astrocytes (**a**–**f**) and neurons (**g**–**r**) in the slices treated with rAbs with or without HC for 48 h. GFAP staining to visualize astrocyte morphology and network. Neuronal cell death was monitored by co-labeling with the dye propidium iodide (PI, *red*) and Purkinje neuron marker Calbindin (*blue*). *Scale bars*: 20 μm

Treatment of slices with MS#30 + HC for 48 h had no overt effect on astrocyte morphology or network structure as visualized with GFAP (glial fibrillary acidic protein) staining. In contrast, NMO#53, which targets AQP4 on astrocytes [2, 33], caused massive disruption of the astrocyte network in the presence of HC (Fig. 4a–f). Purkinje neuron death was monitored by co-labeling with calbindin and propidium iodide (PI). Consistent with previous reports [14], exposure to HC (Iso + HC) induced a low level of Purkinje cell death at 48 h post-treatment. Whereas no notable increase of Purkinje cell death was detected in MS#30 + HC-treated slices when compared to Iso + HC, Purkinje cell death in the slices treated with NMO#53 + HC was significantly elevated as previously described (Fig. 4g–r) [14]. Furthermore, neuronal death was not limited to Purkinje cells, as coincident NeuN and PI staining demonstrated a general loss of neurons throughout the cerebellum of NMO#53 + HC-treated slices, including the granule cell layer [14]. Even with extended treatment (72 h), the morphology and survival of astrocytes and neurons remained unaffected in MS#30 + HC treated slices (data not shown). Thus, in the presence of HC, MS#30 caused significant oligodendrocyte cell loss with no apparent effect on astrocyte and neuronal survival, while NMO#53 treatment killed astrocytes resulting in significant downstream loss of both oligodendrocytes and neurons.

**Fig. 5:**
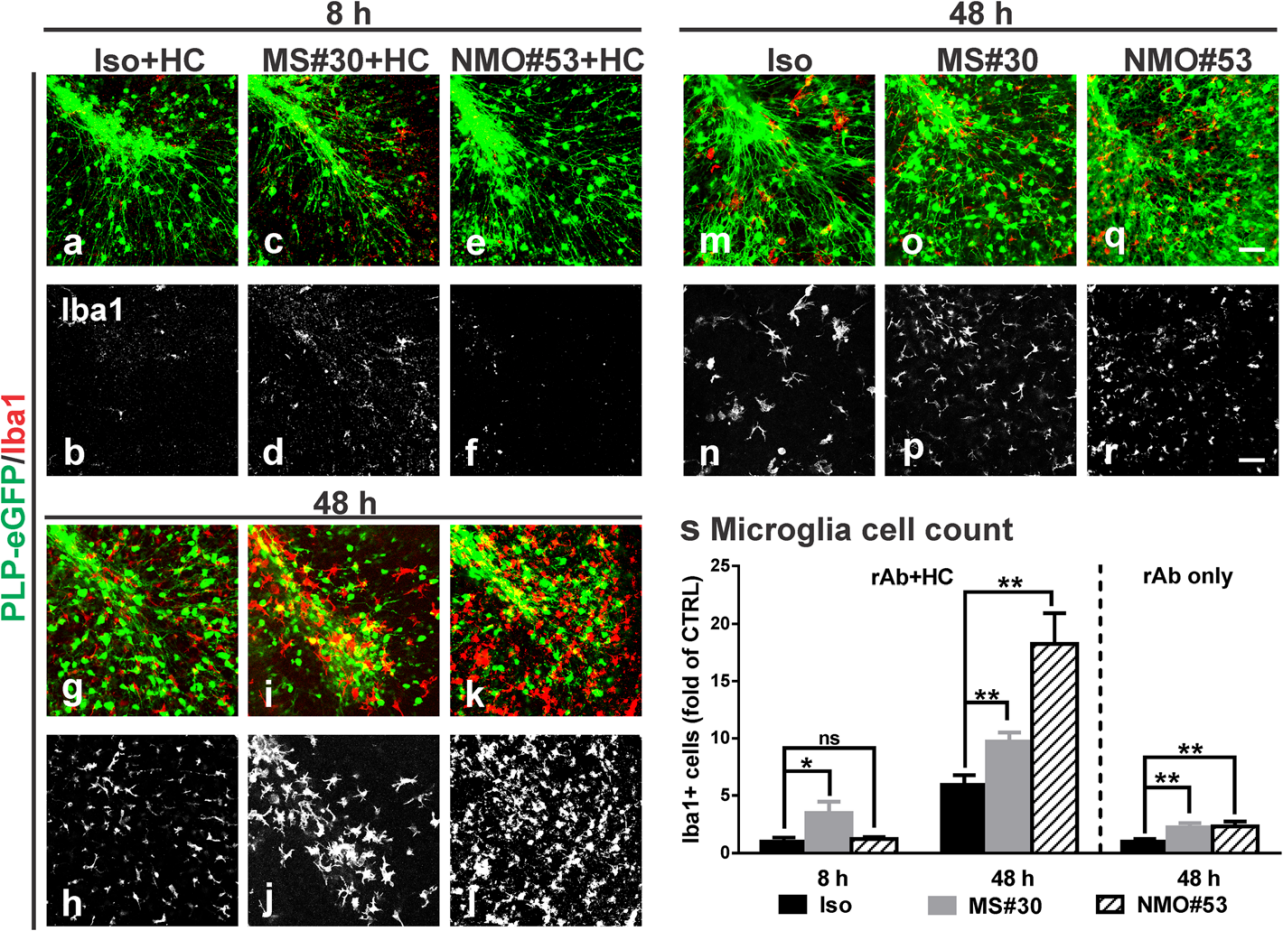
Activation of microglia in MS#30 and NMO#53 treated slices. Cerebellar slices treated with rAbs with (**a**–**l**) or without (**m**–**r**) HC were fixed at indicated time points and then stained with microglia marker Iba1 (*red*). Quantification of Iba1+ cell numbers in slices (**s**). Cell count was normalized to control (*Iso + HC treated slices at 8 h in rAb + HC panel and Iso treated slices at 48 h in rAb only*). Statistical analyses were performed using multiple unpaired Student’s *t* test. *: *p* < 0.05, **: *p* < 0.01, ns: not significant, *n* = 4. *Scale bars*: 50 μm

Microglia activation was assayed by Iba1 (ionized calcium binding adaptor molecule 1) staining (Fig. 5a–r). Increased numbers of Iba1+ microglia were observed around oligodendrocytes in MS#30 + HC-treated slices beginning at 8 h (Fig. 5c, d). Numbers continued to increase with time in focal areas of damage adjacent degenerating oligodendrocytes (Fig. 5i, j, s). In NMO#53 + HC treated slices, microglia activation began later, becoming clearly prominent at the 48 h time point. Increased numbers of robust Iba1+ microglia were dispersed evenly throughout the folia mirroring the broader area of damage occurring within NMO#53 + HC slices (Fig. 5k, l, s). Prolonged treatment with Iso + HC (48 h) also resulted in increased microglia activation with time of exposure, but at levels significantly lower than with MS#30 + HC and NMO#53 + HC (Fig. 5g, h, s). Although treatment with rAbs alone revealed differences in microglia cell numbers between Iso and pathogenic MS and NMO rAbs, the values were far below those observed in the presence of HC (Fig. 5m–r, s).

### Complement activation drives MS#30-mediated oligodendrocyte cytotoxicity

**Fig. 6:**
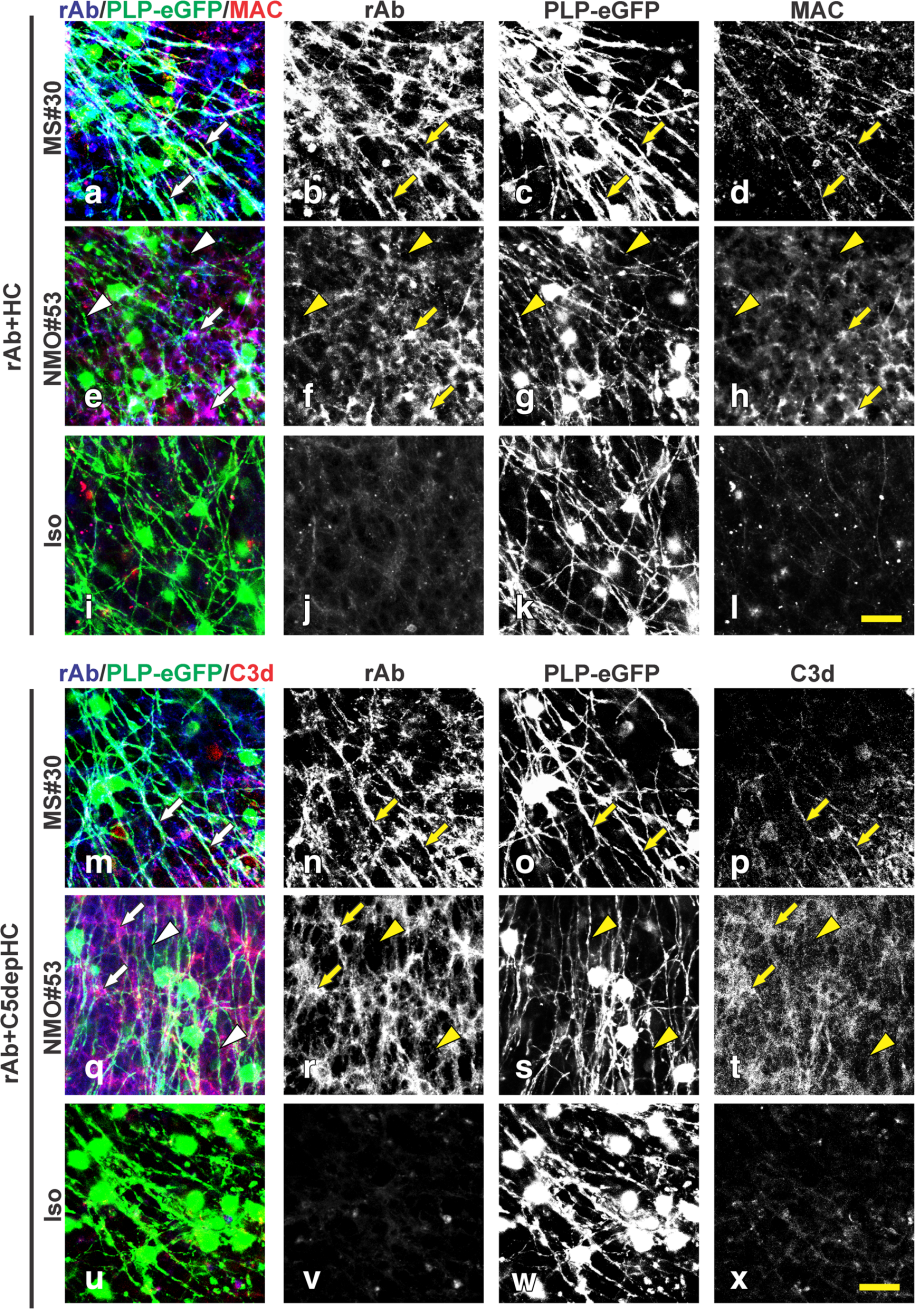
MS#30 and NMO#53 cause cytotoxicity to targeted cells via complement activation. PLP-eGFP slices were treated with rAb + HC (**a**–**l**) or rAb + C5-depleted HC (**m**–**x**) for 8 h and then stained for MAC and C3d deposition, respectively. Deposits of MAC (*red*) or C3d (*red*) were detected along PLP-eGFP+ oligodendrocyte processes and co-localized with MS#30 rAb (*blue*) in MS#30 + HC treated slices (**a**–**d**, **m**–**p**; *arrows*); whereas in NMO#53 + HC treated slices (**e**–**h**, **q**–**t**), MAC was co-localized with NMO#53 rAb (*blue*), which specifically labels astrocytes and end-foot processes (*arrows*), but was absent from oligodendrocyte processes (*arrow heads*). MAC and C3d staining were notably increased in MS#30 + HC or NMO#53 + HC treated slices when compared to Iso + HC treated ones (**i**–**l**, **u**–**x**). *Scale bar*: 25 μm

Based on the requirement for exogenous HC (Figs. 2 and 3), we next examined whether the localization of activated complement components correlated with the distinct patterns of tissue injury initiated by myelin- and AQP4-specific rAbs. To document the presence of terminal complement activation, slices were immune-stained for membrane attack complex (MAC). Deposits of MAC were remarkably enhanced in both MS#30 + HC- and NMO#53 + HC-treated slices, and co-localized with the unique patterns of MS#30 and NMO#53 binding. Following 8 h treatment, MAC staining was detected along oligodendrocyte processes in MS#30 + HC treated slices (Fig. 6a–d; arrows); whereas in NMO#53 + HC-treated slices, MAC was absent from oligodendrocyte processes (Fig. 6e–h; arrow heads), but co-localized instead with NMO#53 rAb on astrocytes (Fig. 6e–h; arrows). Using complement C5-depleted serum, we arrested complement activation following C3 proteolytic cleavage, preventing MAC deposition and complement-mediated lysis, but allowing C3d deposition on complement-targeted cells. C3d deposition was detected on oligodendrocyte processes in MS#30 + C5-depleted HC- treated slices (Fig. 6m–p; arrows), but not in NMO#53 + C5-depleted HC slices (Fig. 6q–t; arrow heads). Instead, C3d was on cells presumed to be astrocytes (Fig. 6q–t; arrows) as previously demonstrated for NMO#53-mediated astrocyte cytotoxicity [14]. These results demonstrate that complement activated by MS#30 or NMO#53 is specifically targeted to oligodendrocytes or astrocytes based on their respective antigenic specificities. Despite a common effector pathway of complement activation, MS#30 and NMO#53 displayed differences in the patterns and kinetics of myelin injury (Fig. 3) and in downstream pathology, indicating that targeting of different neural cell types and not complement activation per se, is the likely key to driving pathologic changes that distinguish NMO and MS demyelination in cerebellar tissue.

### Myelin-binding MS rAbs are less frequent, but present in multiple MS patient CSF

**Fig. 7:**
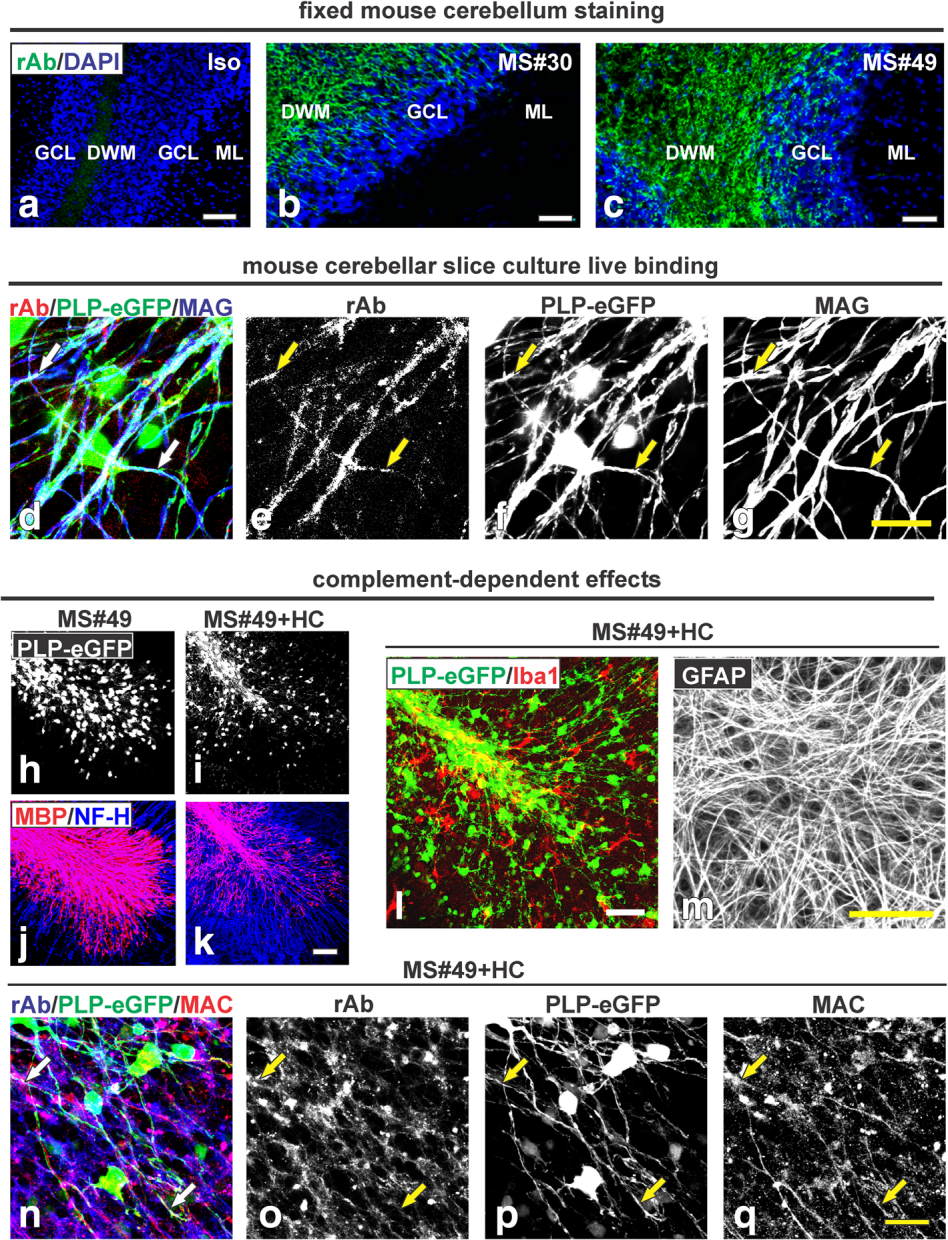
MS rAb#49 (MS#49) binds to myelin and causes demyelination in presence of HC. Staining of mouse cerebellum with isotype control rAb (Iso) is negative (**a**) whereas MS #30 rAb staining (**b**) showed localization in cerebellar deep white matter (DWM) and myelinated tracks within the granule cell layer (GCL). Note the abrupt absence of staining at the molecular layer (ML). A similar staining pattern was observed with MS#49 rAb (**c**). All rAbs (*green*) were used at 20 μg/ml. *Scale bar*: 50 μm. MS#49 bound to oligodendrocyte processes and myelinated MAG+ axons in cultured mouse cerebellar slices (**d**–**g**). Live slices prepared from PLP-eGFP mouse pups were incubated with 20 μg/ml MS#49 rAb (*red*) then fixed and immunostained with anti-human secondary and MAG (*blue*). MS#49 live binding signals were co-localized with PLP-eGFP+ oligodendrocyte processes and MAG+ myelinated axons (stained with NF-H, *arrows*). Scale bar: 10 μm. MS#49 induced complement-dependent cytotoxicity to mature oligodendrocytes and demyelination. Confocal images of PLP-eGFP in slices treated with MS#49 −/+ HC for 48 h (**h**, **i**) and then fixed and stained with MBP (*red*) and NF-H (*blue*) antibodies (**j**, **k**). *Scale bar*: 100 μm. MS#49 + HC induced microglia activation with no effect on astrocytes. PLP-eGFP slices treated with MS#49 + HC for 48 h were fixed and stained with Iba1 (**l**) and GFAP (**m**). *Scale bar*: 50 μm. MS#49 treatment activates the complement pathway on oligodendrocyte processes (**n**–**q**). PLP-eGFP slices were treated with MS#49 + HC for 48 h and stained with MAC (*red*). Deposits of MAC were detected along PLP-eGFP+ oligodendrocyte processes and co-localized with MS#49 rAb (*blue, arrows*). *Scale bar*: 25 μm

Since our initial description of MS#30 binding to myelin [3], we identified additional myelin reactive rAbs from a CIS patient following their first clinical event, who subsequently developed clinically definite MS. The CSF plasmablast clone used to generate the myelin-specific rAb MS#49 represented the largest clone within this patient’s plasmablast repertoire. MS#49 displayed the same binding pattern to white matter tracts in mouse cerebellum as previously described in detail for myelin-binding MS#30 [3]. Staining patterns of both rAbs localized to cerebellar deep white matter and myelinated axons traversing the granule cell layer, but was abruptly absent at the molecular layer (Fig. 7b, c). In live staining of cerebellar slice cultures, MS#49 also bound the surface of eGFP+ oligodendrocyte processes and MAG+ myelinated axons (Fig. 7d–g; arrows). MS#49 demonstrated identical pathology to that observed with MS#30 + HC. In the presence of HC, MS#49 caused oligodendrocyte loss and demyelination, while MS#49 alone had no such effects (Fig. 7h–k). MS#49 plus complement also caused microglia activation (Fig. 7l) with no damage to astrocytes (Fig. 7m). Identical to tissue changes observed after exposure to MS#30 + HC, MAC staining co-localized with MS#49 rAb staining along oligodendrocyte processes in MS#49 + HC treated slices (Fig. 7n–q; arrows), demonstrating targeted oligodendrocyte complement activation.

## Discussion

Our studies demonstrate the presence of myelin-specific plasma blast clones in the CSF of some MS patients. Using structured illumination microscopy and confocal microscopy to assess live cell binding, rAb MS#30 and MS#49 label the surface of oligodendrocyte processes and myelinated axons adjacent to MOG and exterior to MAG. More importantly, these myelin-binding MS rAbs activate the complement pathway and induce robust oligodendrocyte loss and microglial activation, demonstrating their potential to contribute to demyelination in MS patients.

**Fig. 8:**
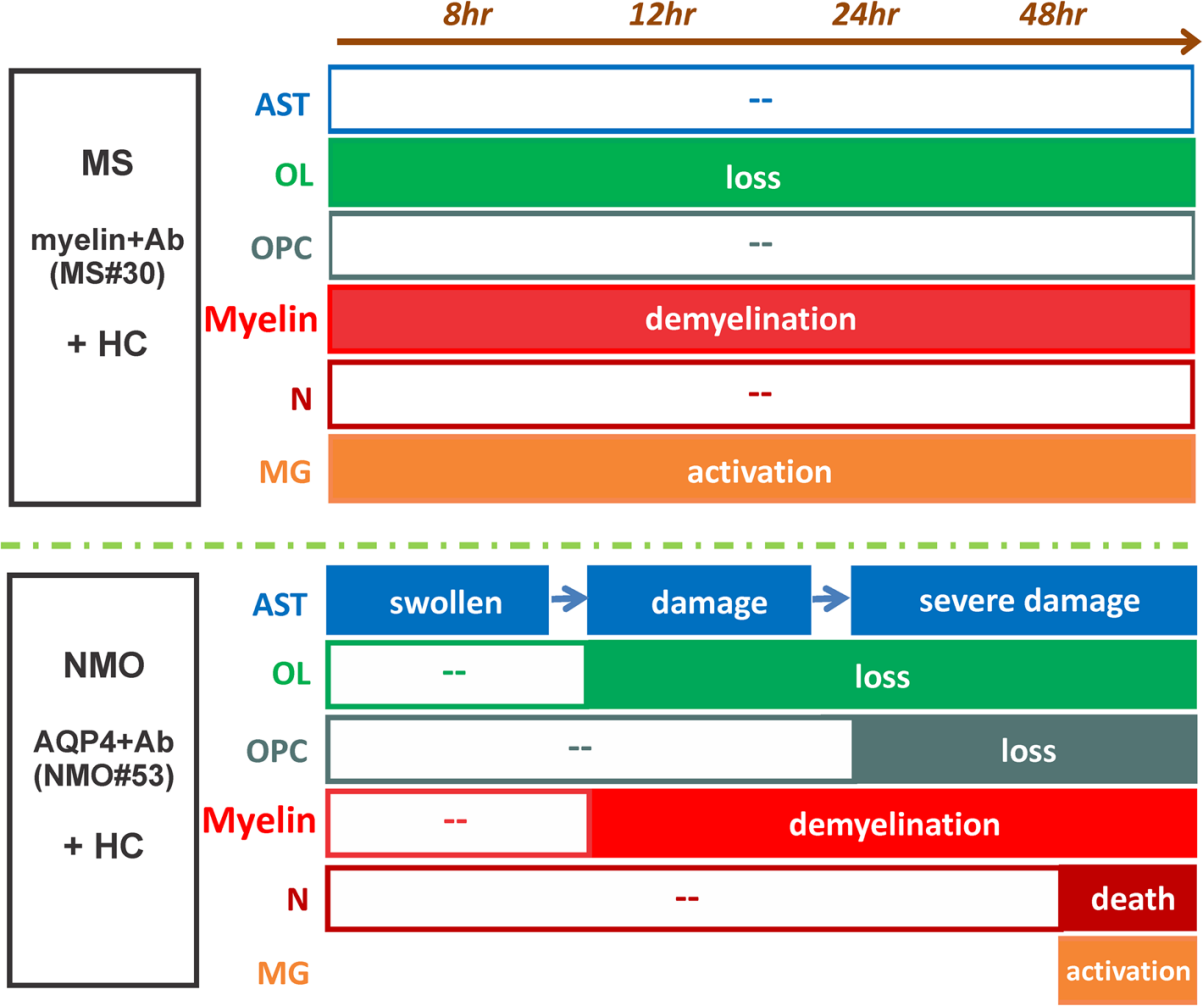
Schematic of distinct effects on glial and neuronal cells induced by MS and NMO antibodies in cerebellar slices. MS myelin-specific (myelin + Ab, such as MS#30 and MS#49) and NMO AQP4-specific (AQP4+ Ab, such as NMO#53) antibodies cause distinct patterns of complement-dependent tissue injury. Beginning at 8 h post-treatment, myelin-specific MS rAb MS#30 targets oligodendrocyte processes and induce rapid and increasing loss of oligodendrocytes, demyelination and microglia activation in the presence of complement (+HC). No significant effects on oligodendrocyte progenitors, neurons or astrocytes are observed. In contrast, NMO AQP4+ rAb targets astrocytes and induces robust complement-dependent astrocyte destruction. At 8 h post-treatment, astrocytes are swollen and the network of astrocytic processes is disrupted. At 48 h, massive destruction of the astrocyte network is apparent. Following astrocyte damage, there is a sequential loss of mature oligodendrocytes, demyelination, oligodendrocyte progenitor reduction, microglia activation and neuronal death [14]. AST: astrocyte, OL: oligodendrocyte, OPC: oligodendrocyte progenitor cell, N: neuron, MG: microglia

In organotypic cerebellar cultures, exposure to myelin-specific rAbs + HC resulted in rapid morphologic changes to oligodendrocytes and their processes accompanied by terminal complement deposition, microglial activation and mature oligodendrocyte cell loss, but with preservation of oligodendrocyte precursors (Figs. 3, 4 and 5). Demyelination induced by myelin-specific MS rAbs was distinct from AQP4 autoantibody-mediated demyelination. Whereas both cerebellar astrocytes and neurons remain intact at 48 h in slices when exposed to myelin-specific MS rAbs, AQP4 autoantibody initiated complement-dependent astrocyte damage, followed by oligodendrocyte cell death, loss of oligodendrocyte precursors [14] and extensive neuronal cell death (Fig. 4). We have previously reported that with ongoing astrocyte destruction induced by AQP4 autoantibody NMO#53 + HC, loss of oligodendrocytes increased from 30% at 12 h to 50% by 48 h. Also, approximately 50% of NG2+ Olig2+ oligodendrocyte precursors were lost in slices by 24 h after treatment with NMO #53 + HC [14]. The damage pattern in this model was thus consistent with *in vivo *murine models of NMO lesion formation that demonstrate initial astrocyte depletion followed by loss of oligodendrocytes and progenitors [32]. Rapid Iba1 upregulation in the presence of myelin-specific rAb plus HC is consistent with the role of microglia as early tissue-resident sensors of CNS injury [20]. Activated microglia were observed in focal areas around degenerating oligodendrocytes in MS#30 + HC-treated slices beginning at 8 h (Fig. 5), whereas in NMO#53 + HC treated slices, microglia activation began later becoming clearly prominent at the 48 h time point and dispersed evenly throughout the folia. Despite early complement-mediated killing of astrocytes by NMO#53, the differences in kinetics of microglia activation in the cerebellar slice cultures between MS#30 and NMO#53 indicates that microglia activation might be independent of complement activation. Instead, microglia may be activated by sensing debris of dead oligodendrocytes and/or damaged myelin, which leads to clearance of debris. Even with extended treatment, the morphology and survival of astrocytes and neurons remained unaffected in slices treated with MS#30 + HC, suggesting that early microglial activation may offer a protective or beneficial effect [27]. The distinct patterns of injury induced by the MS and NMO antibodies in the presence of complement indicate that the target of complement-dependent cytotoxicity, and not activation of the complement cascade itself, is important for delineating the spectrum of glial and neuronal injury (Fig. 8). The important supporting role of astrocytes to both oligodendrocytes and neurons [11] distinguishes damage by NMO antibody from that occurring by direct targeting of oligodendrocyte processes. Our results further distinguish these demyelinating disorders and their mechanisms of oligodendrocyte loss [1, 16, 23, 32, 33].

Extensive investigation has been performed to pathologically characterize and classify MS lesions. The staging system distinguishes active, chronic active, inactive and pre-active lesions [30]. In relapsing-remitting MS, the lesions are often active or chronic active, with axonal transections and massive infiltration and accumulation of inflammatory cells. In the progressive stage of disease, inactive lesions are more typically characterized by axonal loss, astrogliosis and minor infiltration of immune cells [26]. In the majority of active MS lesions, tissue histopathology demonstrates oligodendrocyte apoptosis, deposition of immunoglobulin and terminally activated complement [15] or T cell-mediated as separate and distinct cause of CNS demyelination, whereas others have reported oligodendrocyte apoptosis in the absence of inflammation as the earliest event in lesion development damage [25]. Importantly, our cerebellar slice model recapitulates some of the reported pathologic features of active MS lesions and provides strong evidence that antibodies produced by B cell populations expanded within the CNS compartment have the potential to drive complement-dependent oligodendrocyte cytotoxicity and contribute to lesion formation. Given the absence of a peripheral immune compartment in the cerebellar slice culture model, methodologic restrictions prevent the replication of some seminal features of inflammatory MS lesions, such as myelin phagocytosis, axonal loss and astrogliosis in this slice system. The development of an *in vivo *model of MS rAb antibody injury should allow investigators to further distinguish attributes of antibody-mediated from cell-mediated pathologies.

We have currently cloned myein-specific rAbs from 2 of 4 MS patients analyzed, but their antigenic target remains unknown. Localization of Ab binding to the outer surface of oligodendrocyte processes and myelin suggests a limited possibility of candidate antigens. MS#30 failed to bind to HEK cells expressing the myelin surface protein MOG [22], nor did it bind to the myelin glycolipids sulfatide and galC on lipid arrays [5]. We postulate that higher order multimolecular complexes may be driving antigen specificity. In addition to recognizing myelin-enriched antigens, antibodies cloned from other MS CSF plasmablasts bind to antigens expressed on astrocytes and neurons [3, 13]. It is possible that these antibodies may induce secondary demyelination as observed with AQP4-targeted rAbs [32, 33]. The impact of injured astrocytes and neurons on oligodendrocytes through glia-glia [12, 17] and axon-glia interactions [6, 7, 31] has been well documented. These antibodies may also cause primary degeneration or facilitate the removal of cellular debris.

The results of autologous hematopoietic stem cell transplantation (AHSCT) [19] and therapeutic B cell depletion [8] have called into question the role of antibody in MS lesion formation, despite numerous examples of antibody and complement-mediated demyelination in post-mortem MS autopsy specimens. In autopsy specimens from AHSCT-treated MS patients, extensive demyelination and axonal degeneration are observed in the presence of innate immune activation, but without notable B cell infiltration [19]. Following peripheral B cell depletion, significant reduction in inflammatory lesion formation is observed in the absence of changes in intrathecal IgG synthesis or oligoclonal bands [9, 10]. However, it must be noted that antibody deposition and complement activation were not directly evaluated in post-AHSCT lesions; and antibody-mediated complement cytotoxicity in MS patients may be suppressed following B cell depletion by limited serum complement extravasation into the CNS from the rapid and extensive reduction of blood-brain barrier breakdown.

## Conclusions

In conclusion, we have identified a subset of early MS patients with expanded CSF plasma blast clones specific for antigenic epitopes present on the surface of oligodendrocyte processes and myelin. Our studies using an *ex vivo *slice model reveal that these myelin-specific antibodies drive complement-dependent oligodendrocyte loss and demyelination and could be contributors to type II MS lesions. Whether pathogenic myelin-specific Abs are rare or more common, both within MS CSF plasmablast repertoires and amongst a wider spectrum of MS patients are crucial questions that will require identification of the relevant myelin antigens and the development of assays to screen a large sampling of MS CSF and serum.
